# Therapeutic effects of adipose-derived mesenchymal stem/stromal cells with enhanced migration ability and hepatocyte growth factor secretion by low-molecular-weight heparin treatment in bleomycin-induced mouse models of systemic sclerosis

**DOI:** 10.1186/s13075-022-02915-6

**Published:** 2022-10-07

**Authors:** Takayasu Suzuka, Takuya Kotani, Takashi Saito, Shogo Matsuda, Takako Sato, Tohru Takeuchi

**Affiliations:** 1 Division of Rheumatology, Department of Internal Medicine, Osaka Medical and Pharmaceutical University, Daigaku-Machi 2-7, Takatsuki, Osaka, Japan; 2 Department of Legal Medicine, Osaka Medical and Pharmaceutical University, Daigaku-Machi 2-7, Takatsuki, Osaka, Japan

**Keywords:** Adipose-derived mesenchymal stem/stromal cells, Low-molecular-weight heparin, Hepatocyte growth factor, Systemic sclerosis

## Abstract

**Background:**

Adipose-derived mesenchymal stem cells (ASCs) have gained attention as a new treatment for systemic sclerosis (SSc). Low-molecular-weight heparin (LMWH) enhances cell function and stimulates the production of hepatocyte growth factor (HGF) in a variety of cells. This study investigated the effects of LMWH on the functions of mouse ASCs (mASCs), and the therapeutic effects of mASCs activated with LMWH (hep-mASCs) in mouse models of SSc.

**Methods:**

The cellular functions of mASCs cultured with different concentrations of LMWH were determined. Mice were divided into four groups: bleomycin (BLM)-induced SSc (BLM-alone), BLM-induced SSc administered with mASCs (BLM-mASC), and BLM-induced SSc administered with mASCs activated with 10 or 100 μg/mL LMWH (BLM-hep-mASC); there were 9 mice per group (*n* = 9). Skin inflammation and fibrosis were evaluated using histological and biochemical examinations and gene expression levels.

**Results:**

In vitro assays showed that migration ability and HGF production were significantly higher in hep-mASCs than in mASCs alone. The mRNA expression levels of cell migration factors were significantly upregulated in hep-mASCs compared to those in mASCs alone. The hep-mASCs accumulated in the skin tissues more than mASCs alone. The thickness of skin and hydroxyproline content in BLM-hep-mASC groups were significantly decreased, and the skin mRNA expression levels of interleukin-2, α-smooth muscle actin, transforming growth factor β1, collagen type 1 alpha 1, and tissue inhibitor of metalloproteinase 2 were significantly downregulated compared to those in the BLM-alone group.

**Conclusions:**

hep-mASCs showed higher anti-inflammatory and anti-fibrotic effects than mASCs alone and may be a promising candidate for SSc treatment.

**Supplementary Information:**

The online version contains supplementary material available at 10.1186/s13075-022-02915-6.

## Background

Systemic sclerosis (SSc) is a refractory autoimmune disease that causes inflammation, fibrosis, and vascular endothelial damage in systemic organs [1, 2]. The main skin symptoms of SSc are cutaneous sclerosis and skin ulcer, which affect the daily activities of the patients and decrease the quality of their life [1, 2]. There is no established treatment for skin lesions in patients with SSc. In many cases, SSc has been empirically treated with a combination therapy of corticosteroid and oral cyclophosphamide, intravenous cyclophosphamide, or mycophenolate mofetil [3–5]. Anti-fibrotic drugs—such as pirfenidone and nintedanib—are reported to suppress the decline in forced vital capacity in patients with idiopathic pulmonary fibrosis [6, 7], and SSc-associated interstitial lung disease [8]. However, the effect of these treatments on scleroderma in patients with SSc is limited. In addition, the drug toxicity resulting from immunosuppressive therapy causes side effects—such as infection, bone marrow (BM) suppression, subsequent malignancy, and organ damage. Thus, there is a need to develop better treatments in terms of efficacy and tolerability for skin lesions in patients with SSc.

Mesenchymal stem cells (MSCs) are being studied extensively in regenerative medicine because of their ability to differentiate into a variety of mesenchymal cells—including osteoblasts, adipocytes, myocytes, and chondrocytes [9, 10]. These cells have anti-apoptotic, anti-inflammatory, and anti-fibrotic effects, as well as the ability to modulate the immune response and modify the microenvironment at the site of engraftment [11, 12]. The MSCs can also be used as allografts because they express low levels of human leukocyte antigen classes I and II [11–13] and are well tolerated when administered intravenously [14]. Apart from BM, recent studies show that MSCs can be harvested from other tissues—including cord blood, placenta, and adipose tissue. The adipose-derived mesenchymal stem cells (ASCs) have gained much attention as the adipose tissue contains large numbers of MSCs, and subcutaneous adipose tissue is easily accessible.

ASCs are superior to other types of MSCs—including BM-MSCs in immunomodulatory effect: suppressing both the proliferation of T cells and the differentiation of monocytes into dendritic cells towards maturity [15], and in inhibiting the activation and immunoglobulin production of B cells [16, 17]. Furthermore, ASCs are more potent than other MSCs in terms of their pro-angiogenic, anti-apoptotic, and anti-oxidative effects [15, 18, 19]. Collectively, these findings indicate that ASCs may be more effective than other types of MSCs [20].

Heparin is an inhibitor of antithrombin III and factor Xa and is used in the prevention and treatment of thrombosis [21]. It interacts with many proteins and is expected to have multifaceted efficacy [22]. Heparin stabilises HGF dimers to promote dimerisation and activation of the c-Met receptor [23]. It enhances fibroblast growth factor and bone morphogenetic protein 4 gene expression, increasing proliferation and pluripotency in BM-MSCs and embryonic stem cells, respectively [24–26]. Heparin stimulates the biosynthesis of HGF in various cells, such as lung fibroblasts, promyelocytic leukaemia cells, and umbilical vein endothelial cells—the detailed mechanism of this stimulation is unknown [27, 28]. Normal heparin accelerates bleeding [29] whereas low-molecular-weight heparin (LMWH) can induce less bleeding [30]. In addition, the ability of LMWH to secrete HGF is similar to that of normal heparin [31].

This study tested the hypothesis that LMWH-activated ASCs would exhibit a synergistically favourable effect on SSc by promoting anti-inflammatory and anti-fibrotic effects. The effects of LMWH on the functions of ASCs were investigated, and the therapeutic effect of LMWH-activated ASCs was compared with that of ASCs alone in mouse models of SSc.

## Materials and methods

### Adipose tissue harvesting, isolation of ASCs, and their culture

The Institutional Animal Care and Use Committee of Osaka Medical and Pharmaceutical University approved all of the following research protocols (approval ID: 21050-A), including the surgical procedures and animal care, and all methods were performed in accordance with the relevant guidelines and regulations. Mice were maintained under a specifically controlled pathogen-free environment (temperature, 25°C; humidity, 50–70%; light/dark cycle, 12 h/12 h) with free access to food and water. Female 8-week-old Balb/c mice (SHIMIZU Laboratory Supplies, Kyoto, Japan) were sacrificed by cervical dislocation under isoflurane anaesthesia. Adipose tissue was harvested from the inguinal areas and used in all experiments as subcutaneous adipose tissue. Mouse ASCs (mASCs) were isolated from each adipose tissue sample (as previously described) with minor modifications [32]. Briefly, adipose tissue was placed in a collection tube, washed in phosphate-buffered saline (PBS) (pH 7.4) and cut into 0.5–1.0 mm small fragments using fine scissors. The tissues were then transferred into 15-mL tubes. Type I collagenase (1.0 mg/mL in 1% BSA/HBSS(+)) was then added to the tube at an identical volume. The mixture was immediately agitated using a water bass shaker (150 rpm) at 37 °C for 30 min. The digested tissue was filtered through a 40-μm cell strainer and centrifuged at 200×*g* for 5 min. The supernatant was aspirated and the cell pellet was resuspended in erythrocyte lysis buffer (168 mM NH_4_Cl, 10 mM KHCO_3_, and 0.1 mM EDTA-4Na; 10 mL) at 4 °C for 10 min. After erythrocyte lysis, 5 mL of medium was added, and the tube was centrifuged at 200×*g* for 5 min. The cell pellet was resuspended in medium and filtered through sterilised 100- and 40-μm cell strainers (Corning Inc., NY, USA). The mASCs were cultured and used in the third passage.

### Cell proliferation assay

The mASCs were seeded into collagen-coated wells of a 96-well plate (Corning Inc.) at a density of 5000 cells/100 μL per well. They were cultured in DMEM/F-12 containing 10% FBS and 1.0% Pen-Strep for 24 h at 37 °C in a 5% CO_2_/95% air atmosphere. The medium was changed to a medium supplemented with LMWH at 0, 10, and 100 μg/mL, respectively, and cultured for 24 h at 37 °C. The medium was subsequently exchanged with 100 μL of fresh medium containing WST-8 of Cell Count Reagent SF (10 μL) and incubated for 30 min. The absorbance of the medium was measured at 450 nm wavelength. Cell viability was expressed as a percentage compared with cells cultured in DMEM/F-12 containing 10% FBS and 1.0% Pen-Strep. This experiment was repeated five times for each sample.

### Cell migration assay

The migration activity of mASCs was evaluated using a modified Boyden chamber method. mASCs (50,000 cells per well) were seeded into the upper chambers of 24-well culture plates, and the lower chambers were filled with DMEM/F-12 medium containing 2% FBS and 1.0% Pen-Strep supplemented with LMWH at 0, 1, 10, and 100 μg/mL, respectively, followed by incubation for 6 h at 37 °C. The migrated cells were stained with 4’,6-diamidino-2-phenylindole (DAPI) and counted in three randomly selected high-power fields (HPFs: ×200, 0.15 mm^2^ per HPF) per chamber under a fluorescence microscope, and the resulting numbers were averaged. The experiments were independently performed five times for each sample.

### HGF immunoassay

The HGF content of the supernatants was evaluated using an HGF ELISA Quantikine Kit (R&D Systems Inc., MN, USA) in accordance with the protocol of the manufacturer. The data were normalised to the relative cell numbers obtained from the cell proliferation assay. The experiments were carried out six times independently for each sample and in duplicate.

### Quantitative real-time reverse transcription-PCR (RT-qPCR) using mASCs

Total RNA was extracted from mASCs cultured in DMEM/F-12 containing 2% FBS and 1.0% Pen-Strep supplemented with LMWH at 0, 1, 10, or 100 μg/mL. After RNA extraction with an RNeasy mini kit (Qiagen Ltd., Manchester, UK), cDNA was synthesised using an ExScript RT kit (Takara, Shiga, Japan) and amplification was performed in a Sequence Detection System 7000 (Applied Biosystems) according to the manufacturer’s instructions. The primer sequences for stromal derived factor-1 (SDF-1), C-X-C chemokine receptor (CXCR) type 4 (CXCR-4), CXCR type 7 (CXCR-7), and glyceraldehyde 6-phosphate dehydrogenase (GAPDH) are summarised in Table 1. Each target gene expression was normalised to the housekeeping gene expression. The experiments were repeated in triplicate, and results were averaged.

**Table 1:** Primers used in the RT-qPCR analyses *in vitro*

Gene	Forward	Reverse
*CXCR-4*	5′-TCATCAAGCAAGGGTGTGAG-3′	5′-GGCTCCAAGGAAAGCATAGA-3′
*CXCR-7*	5′-AGAAGATGGTACGCCGTGTCG-3′	5′-TCTTCCGGCTGCTGTGCTTCTC-3′
*SDF-1*	5′-AGCCATGTTGCCAGAGCCAACG-3′	5′-CACACACACACCTGGTCCTCATGG-3′
*GAPDH*	5′-ACAATGAATACGGCTACAG-3′	5′-GGTCCAGGGTTTCTTACT-3′

### Animals and experimental groups

For inducing skin fibrosis, the back skins of 8-week-old female BALB/c mice were shaved, and for 21 days, the mice were daily subcutaneously injected with 100 μg/100 μL bleomycin (BLM) (Nippon Kayaku, Tokyo, Japan) in sterile saline [33] (Additional file 1). The mASCs activated with LMWH (hep-mASCs) were cultured in DMEM/F-12 containing 10% FBS and 1.0% Pen-Strep supplemented with LMWH (10 and 100 μg/mL). In this study, to assess the effect of hep-mASCs, mice were assigned to the following groups, with *n* = 9 in each group: BLM-induced SSc (BLM-alone), BLM-induced SSc administered mASCs (BLM-mASC), BLM-induced SSc administered mASCs activated with 10 μg/mL LMWH (BLM-hep10-mASC), and BLM-induced SSc administered mASCs activated with 100 μg/mL LMWH (BLM-hep100-mASC). The mASCs cultured with or without LMWH were intravenously injected into the tail vein with 100 μL of PBS. In the BLM-alone group, 100 μL of PBS was intravenously injected into the tail vein. The number of administered cells was 2.5 × 10^4^ cells. All three treatments were performed on day 7 following BLM administration. The mice were euthanised and their skins were harvested at 21 days after administering BLM.

### Histological analysis

The back skins of each mouse were harvested and fixed for 6 h in 4% PFA/PBS followed by overnight incubation in 20% sucrose/PBS. The tissues were embedded in optimal cutting temperature compound (Sakura FineTek, Tokyo, Japan), cut into 5-μm sections, and stained with haematoxylin and eosin (H&E) or Masson’s trichrome stain. The thickness of the dermis was measured to evaluate skin sclerosis. The distance between the epidermal–dermal junction and the dermal–fat junction was measured at 10 randomly selected sites per section and was averaged.

### Hydroxyproline assay

Hydroxyproline content was determined by measuring the skin tissue. The injected skin sites of each BLM-SSc mouse were punch-biopsied (6 mm) and stored at −70 °C. Each sample was treated with 0.5 mol/L acetic acid-containing pepsin (0.3 mg/10 mg) at 4 °C for 24 h. The hydroxyproline content of the skin tissue was measured using a Sircol^TM^ soluble collagen assay kit (Biocolor Ltd, Northern Ireland).

### Fluorescent immunocytochemistry

The skin sections were washed with PBS, and the samples were blocked in an antibody dilution buffer of 2% BSA/PBS for 15 min at room temperature (RT). After the blocking solution was removed, primary antibodies/markers were added to the antibody dilution buffer at 37 °C for 2 h: anti-CD3 (1:200) (eBioscience, San Diego, CA, USA) for T lymphocytes; anti-F4/80 antibody (1:200) (eBioscience) for macrophages. After washing with PBS, cells were incubated for 30 min at RT with secondary antibodies prepared at 1:500 in antibody dilution buffer: Alexa 594 donkey anti-rat IgG (Jackson ImmunoResearch Laboratories, Inc., West Grove, PA, USA). After the secondary antibodies were removed and the tissues had again been washed with PBS, nuclear counterstaining was performed by incubating with 4’,6-diamidino-2-phenylindole (DAPI) solution (1 μg/mL in PBS; Fujifilm Wako Japan K.K.) for 10 min at RT. A mounting medium (ImmunoBioScience, Mukilteo, WA) was added to the sample slides before they were covered with cover slips and sealed with nail varnish. They were then evaluated under a fluorescence microscope (BZx-700, Keyence, Osaka, Japan). The positively stained cells in each sample were counted in five different high-power fields (×200).

### Quantitative real-time RT-PCR using skin in vivo

Total RNA was extracted from skin tissues harvested at 21 days after BLM administration. Following RNA extraction with an RNeasy fibrous tissue mini kit (Qiagen Ltd.), cDNA was synthesised and amplified. The primer sequences for interleukin-2 (IL-2), interferon-gamma (INF-γ), interleukin-4 (IL-4), interleukin-10 (IL-10), interleukin-13 (IL-13), interleukin-17 (IL-17), interleukin-6 (IL-6), α-smooth muscle actin (α-SMA), transforming growth factor β1 (TGF-β1), collagen type 1 alpha 1 (COL1α1), tissue inhibitor of metalloproteinase 2 (TIMP-2), and GAPDH are summarised in Table 2. The primers for gremlin-1 (GREM-1) were purchased from Sino Biological, Beijing, China. The experiments were repeated in triplicate, and the results were averaged.

**Table 2:** Primers used in the RT-qPCR analyses

Gene	Forward	Reverse
*IL-2*	5′-TGTGTAGGTAGACTCATTA-3′	5′-TTAGAGGAGAGCTTTATTTC-3′
*INF-γ*	5′-TTAACTCAAGTGGCATAG-3′	5′-TGATTCAATGACGCTTAT-3′
*IL-4*	5′-TTAGCATCTCTTGATAAACT-3′	5′-ATATGGCTCCTGGTACAT-3′
*IL-10*	5′-CTATTTAGAAAGAAGCCCAAT-3′	5′-CCCTCCCATCATATAATATAATC-3′
*IL-13*	5′-GTAGCCCACTTTATAACAAA-3′	5′-GGTCTCTCCTCATTAGAAG-3′
*IL-17A*	5′-ACGTTTCTCAGCAAACTTAC-3′	5′-CCCCTTTACACCTTCTTTTC-3′
*IL-6*	5′-AAATGAGAAAAGAGTTGTG-3′	5′-TTTGTATCTCTGGAAGTTT-3′
*α-SMA*	5′-TAAGGCCAACCGGGAGA-3′	5′-AGAGGCATAGAGGGACAGCA-3′
*TGF-β1*	5′-GACATCTCACACAGTATA-3′	5′-GTTGCTATATTTCTGGTAG-3′
*COL1A1*	5′-AAGAAGACATCCCTGAAG-3′	5′-ATACAGATCAAGCATACCT-3′
*TIMP-2*	5′-CTCGGAGCGCAATAAAACGG-3′	5′-CCTCTTGATGGGGTTGCCAT-3′
*GAPDH*	5′-ACAATGAATACGGCTACAG-3′	5′-GGTCCAGGGTTTCTTACT-3′

### Western blotting using skin

The skin tissues were crushed into pieces using an automill machine (2 sets of 2 min at 1500 rpm) in RIPA buffer supplemented with protease inhibitor: PMSF (0.1 mM) and phosphatase inhibitor: sodium fluoride (5 mM).

The protein concentration was analysed using Qubit protein assay kit (Thermo Fisher Scientific, Cleveland, OH, USA).

Western blotting was performed using the Protein Simple WES System (Protein Simple, SanJose, CA, USA) following the protocols given by the manufacturer. Primary antibodies: anti-Smad2/3 (1:20) (Cell Signaling Technologies, Danvers, MA, USA), anti-Phospho-Smad2/3 (1:20) (Cell Signaling Technologies), and secondary antibodies: Anti-Rabbit Secondary HRP Antibody (Protein Simple) were used the western blot. Protein expression was normalized to total protein using the Total Protein Detection Module (Protein Simple). Simple Western data was analysed using Compass Software version 3.1 (Protein Simple).

### Bioluminescence imaging for in vivo analysis of mASCs

The mASCs were incubated with indocyanine green (ICG) in PBS (25 μM) for 30 min at 37 °C followed by rinsing thrice with PBS. The in vivo migration ability and distribution of the hep-mASCs were investigated. The ICG-labelled mASCs—cultured with LMWH (0, 10, and 100 μg/mL)—were intravenously injected into nine mice after a week of BLM administration. Twenty-four hours after administering mASCs, the mice were sacrificed and their skin, thymus, spleen, lungs, heart, and kidneys were harvested. The cell accumulation in each tissue was evaluated as signal intensities using an IVIS Imaging System 200 Series (Calliper Life Science, Hopkinton, MA, USA), and the differences in average signal intensities depending on the LMWH concentration was examined.

### Statistical analysis

All values are presented as mean ± standard error of mean (SEM). Comparisons between two groups were tested using the Mann-Whitney *U* test, and those among multiple groups were tested for significance via analysis of variance (ANOVA) followed by post hoc testing with a Tukey procedure. Statistical significance was set at *P* < 0.05. Statistical analyses were performed using commercially available software (GraphPad Prism, MDF Co. Ltd., Tokyo, Japan).

## Results

### LMWH increased the cellular functions of mASCs in vitro

**Fig. 1:**
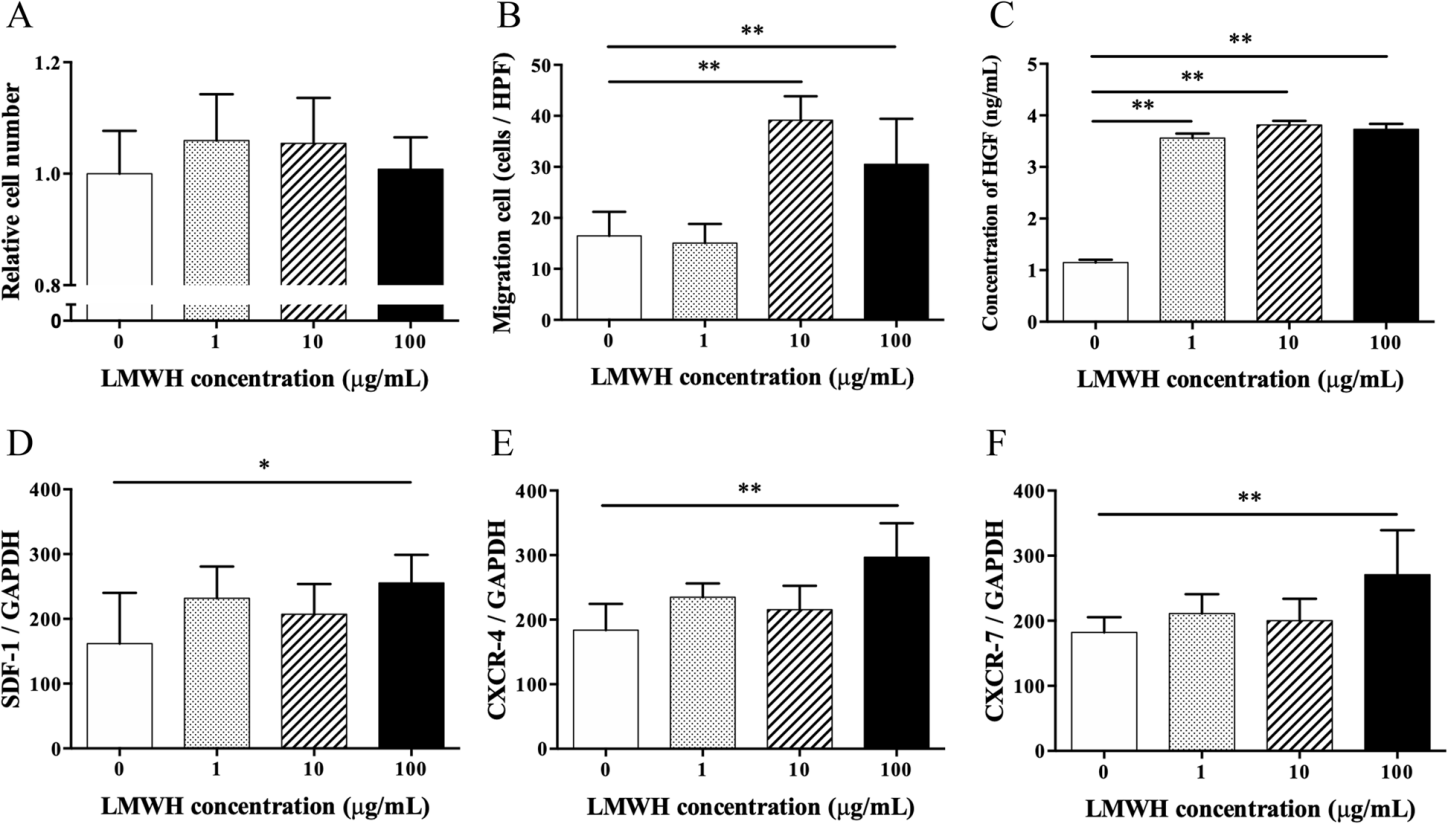
Cellular functions of mASCs from Balb/c mice activated with different concentrations of LMWH. **A** The migration activity of mASCs with 10 and 100 μg/mL LMWH was significantly increased and that with 1 μg/mL LMWH was not increased. **B** The proliferation activity of mASCs in various concentrations of LMWH showed no significant difference. **C** The amounts of HGF secreted by mASCs with 1, 10, and 100 μg/mL LMWH were significantly increased. **D–F** The mRNA expression of chemokine SDF-1, CXCR-4, and CXCR-7 were significantly upregulated in mASCs with 100 μg/mL LMWH, and not in mASCs with 1 and 10 μg/mL LMWH. Data are shown as mean ± standard error of mean (SEM). *P < 0.05; **P < 0.01 vs. mASCs without LMWH

The cellular functions of mASCs from BALB/c mice were assessed in the presence of LMWH. There was no significant difference in the proliferation activity between the LMWH concentrations (Fig. 1A). The migration activity of mASCs with 10 and 100 μg/mL LMWH was significantly increased (that with 1 μg/mL LMWH did not increase) when compared to mASCs without LMWH (Fig. 1B). The amount of HGF secreted by mASCs with 1, 10, and 100 μg/mL LMWH was significantly increased when compared to that without LMWH (Fig. 1C). The mRNA expression of chemokine stromal cell-derived factor-1 (SDF-1), C-X-C chemokine receptor type 4 (CXCR-4), and CXCR-7—cell migration-related factors—was significantly upregulated in mASCs with 100 μg/mL LMWH (except in mASCs with 1 and 10 μg/mL LMWH) when compared to those in mASCs without LMWH (Fig. 1D–F).

### Examination of accumulation of hep-mASCs in each organ by IVIS in BLM-SSc mice

**Fig. 2:**
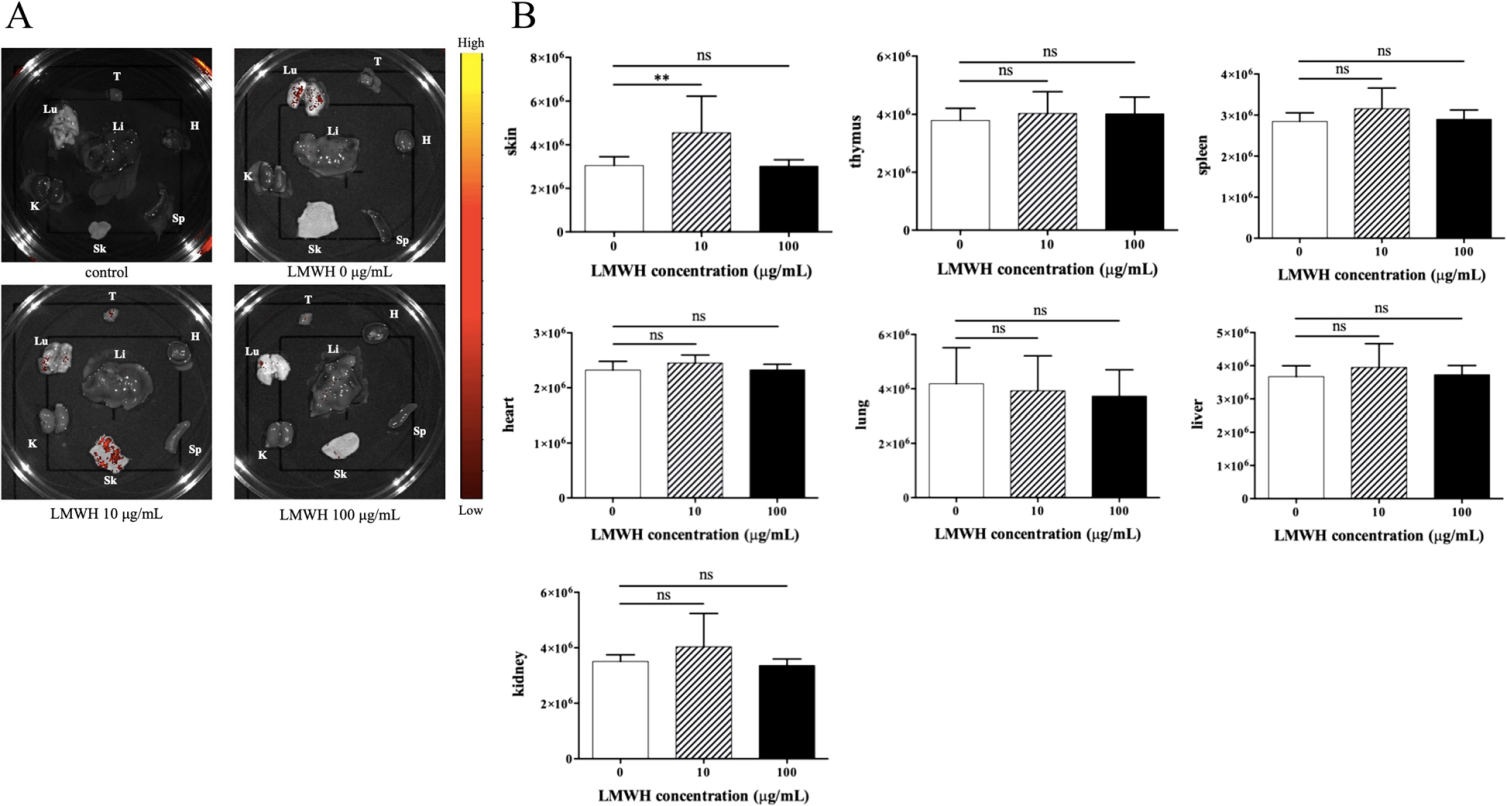
In vivo migration of mASCs cultured in different LMWH concentrations was assessed by IVIS system. **A** Representative accumulation image of ICG-labelled mASCs in each organ after 24 h of mASC administration. The images are displayed with a minimum-maximum scale of 4 × 10^6^ to 2 × 10^7^ photons/s/cm^2^/steradian. **B** The signal intensities in the skin were significantly increased in mASCs with 10 μg/mL LMWH, but there was no significant difference in mASCs with 100 μg/mL LMWH. For the signal intensities in the spleen, thymus, heart, lungs, liver, and kidneys, there was no difference. Data are shown as mean ± SEM. **P < 0.01 vs. mASCs without LMWH. Sk, skin; T, thymus; Sp, spleen; H, heart; Lu, lungs; Li, liver; K, kidneys

The in vivo migration of mASCs cultured with 10 and 100 μg/mL of LMWH was assessed by labelling with ICG and using the IVIS system. Figure 2A shows the accumulation of ICG-labelled mASCs in each organ at each LMWH concentration. Twenty-four hours after administering mASCs, the signal intensities were significantly increased in mASCs with 10 μg/mL of LMWH when compared to those in mASCs without LMWH, but there was no significant difference observed in mASCs with 100 μg/mL of LMWH. For the signal intensities in the spleen, thymus, heart, lungs, liver, and kidneys, there was no difference in mASCs with LMWH when compared to mASCs without LMWH (Fig. 2B).

### Hep-mASCs effectively reduced skin fibrosis in BLM-SSc mice

**Fig. 3:**
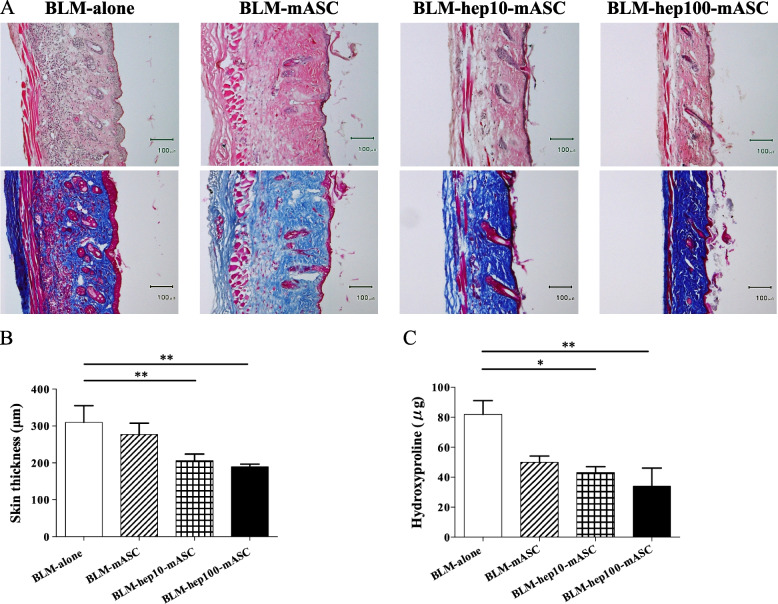
Skin fibrosis reduced after administering hep-mASCs. **A** Representative skin lesion with haematoxylin eosin staining and Masson’s trichrome staining (×100) at 21 days after starting BLM administration. The increase in collagen deposition in the skin was reduced in the BLM-hep10-mASC group and the BLM-hep100-mASC group, but was not reduced in the BLM-mASC group. **B** The thickness of the dermis (distance between the epidermal–dermal junction and the dermal–fat junction) was significantly decreased in the BLM-hep10-mASC group and the BLM-hep100-mASC group, but it did not decrease in the BLM-mASC group. **C** The levels of hydroxyproline contents in the BLM-hep-mASC group were significantly decreased, and those in BLM-mASC group were not decreased. The levels of hydroxyproline contents were significantly decreased in the BLM-hep10-mASC group and the BLM-hep100-mASC group, but it did not decrease in the BLM-mASC group. *n* = 7 in each group. In the BLM-alone group, 100 μL of PBS was intravenously injected into the tail vein. Data are shown as mean ± SEM. *P < 0.05; **P < 0.01 vs. BLM-alone group

Increased collagen deposition caused fibrosis in BLM-SSc skin, which was reduced by administering hep-mASCs and not by administering mASCs without LMWH (Fig. 3A). The thickness of the skin fibrosis was significantly decreased in the BLM-hep10-mASC group and the BLM-hep100-mASC group when compared to the BLM-alone group, but it did not decrease in the BLM-mASC group (Fig. 3B). Collagen deposition in the skin was biochemically assessed by measuring the hydroxyproline content. The hydroxyproline levels in the BLM-hep10-mASC group and the BLM-hep100-mASC group were significantly lower than those in the BLM-only group, but there was no difference in the BLM-mASC group (Fig. 3C).

### Hep-mASCs reduced infiltration of inflammatory cells in the skin fields of BLM-SSc mice

**Fig. 4:**
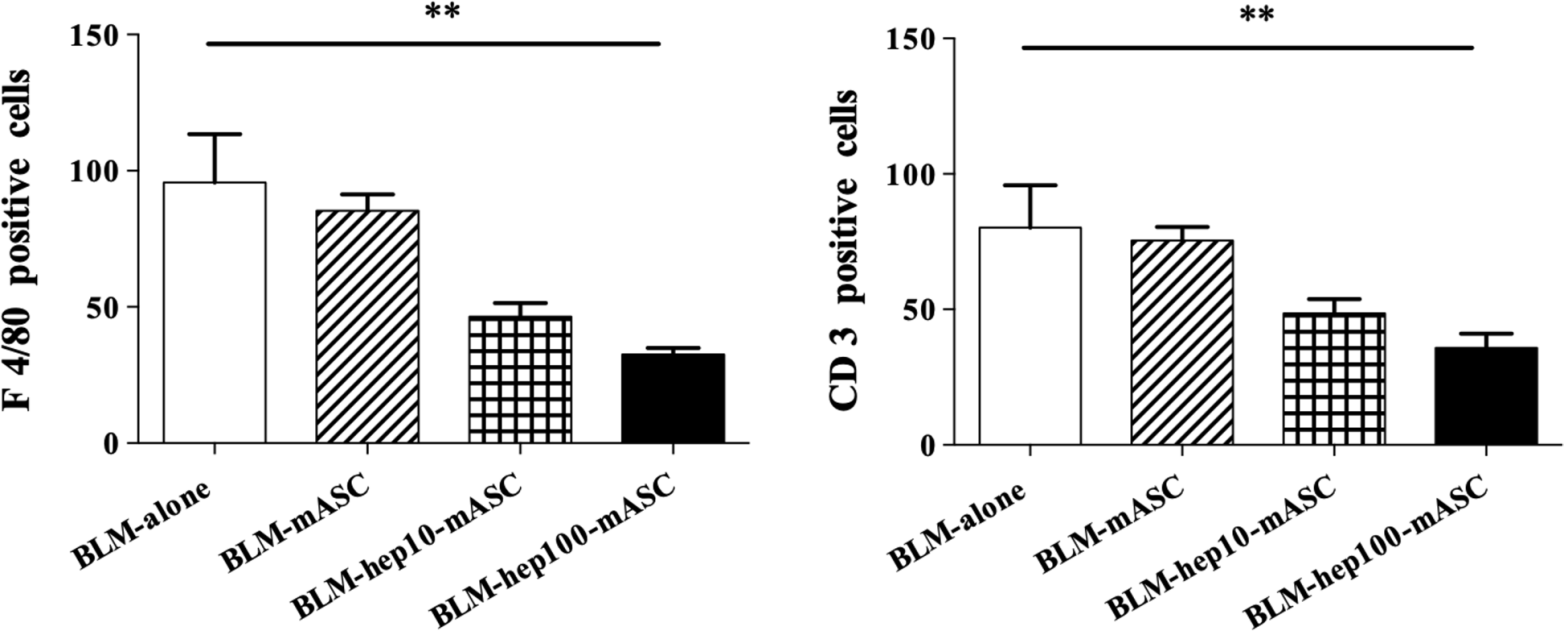
Infiltration of inflammatory cells in the skin were reduced by administering hep-mASCs. In comparison to the BLM-alone group, the infiltration of macrophages and T lymphocytes in the BLM-hep10-mASC group tended to be reduced, and those in the BLM-hep100-mASC group were even more significantly reduced. *n* = 7 in each group. Data are shown as mean ± SEM. **P < 0.01 vs. BLM-alone group

In comparison to the BLM-alone group, the infiltration of macrophages and T lymphocytes in the BLM-hep10-mASC group tended to be reduced, and those in the BLM-hep100-mASC group were even more significantly reduced (Fig. 4).

### Expression levels of genes related to tissue inflammation in the skin of BLM-SSc mice were regulated by administering mASCs activated with LMWH

**Fig. 5:**
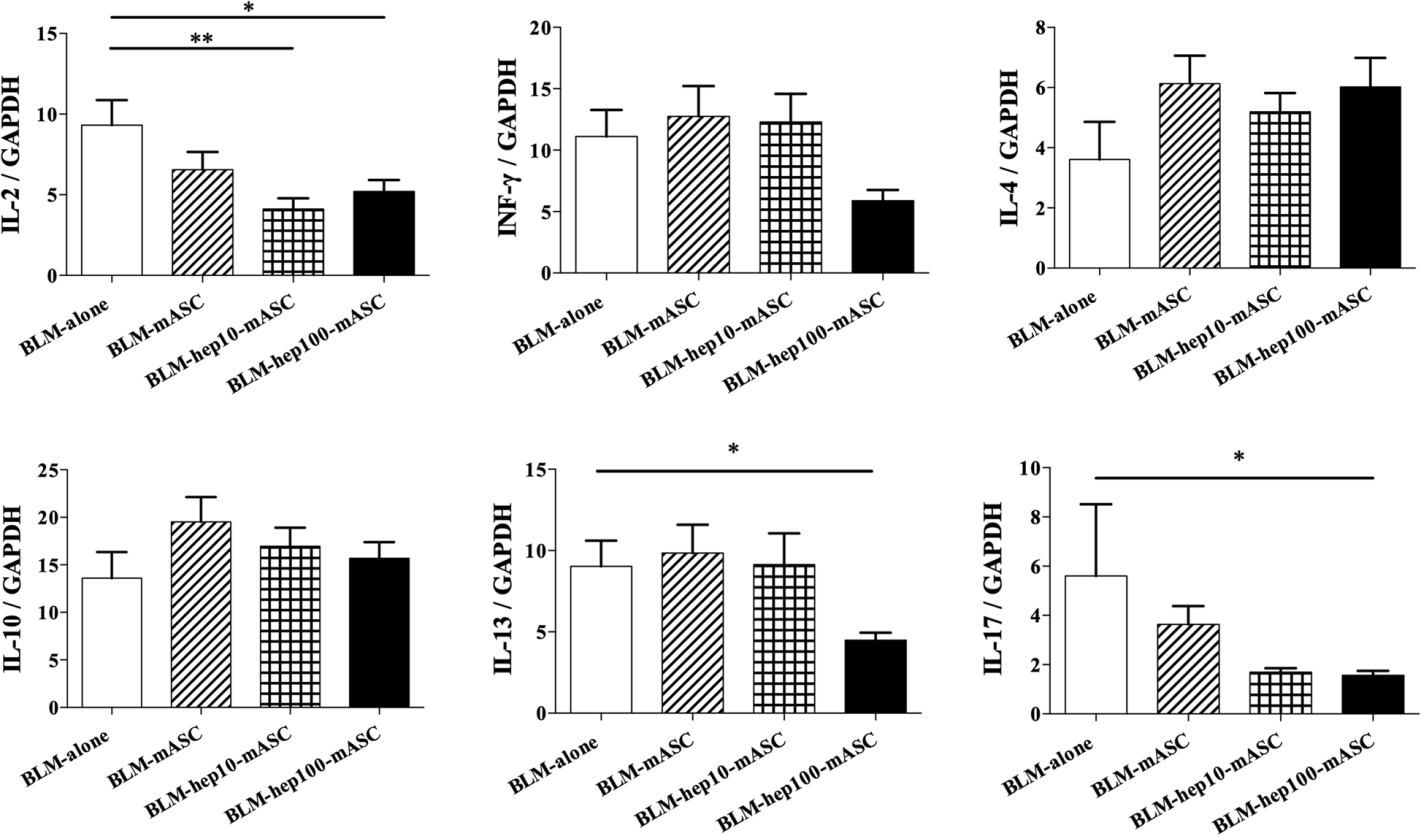
Genes related to inflammation in skin were regulated by administering hep-mASCs. The skin mRNA expression levels of IL-2 were significantly downregulated in the BLM-hep10-mASC group and the BLM-hep100-mASC group compared to those in the BLM-only group. The skin mRNA expression levels of IL-13 and IL-17 were significantly downregulated in the BLM-hep100-mASC group compared to those in the BLM-only group. *n* = 7 in each group. Data are shown as mean ± SEM. *P < 0.05; **P < 0.01 vs. BLM-alone group

To evaluate the anti-inflammatory effects of hep-mASCs, skin mRNA expression was analysed by qRT-PCR. The skin mRNA expression levels of IL-2 were significantly downregulated in the BLM-hep10-mASC group and the BLM-hep100-mASC group compared to those in the BLM-only group, but were not downregulated in the BLM-mASC group. Compared to the BLM-only group, the skin mRNA expression levels of IL-13 and IL-17 were significantly downregulated in the BLM-hep100-mASC group, but were not downregulated in either the BLM-mASC group or the BLM-hep10-mASC group. There was no significant different in skin mRNA expression levels of INF-γ, IL-4, IL-10, and IL-6 between the groups (Fig. 5, Additional file 2).

### Expression levels of genes related to tissue fibrosis in the skin of BLM-SSc mice were regulated by administering mASCs activated with LMWH

**Fig. 6:**
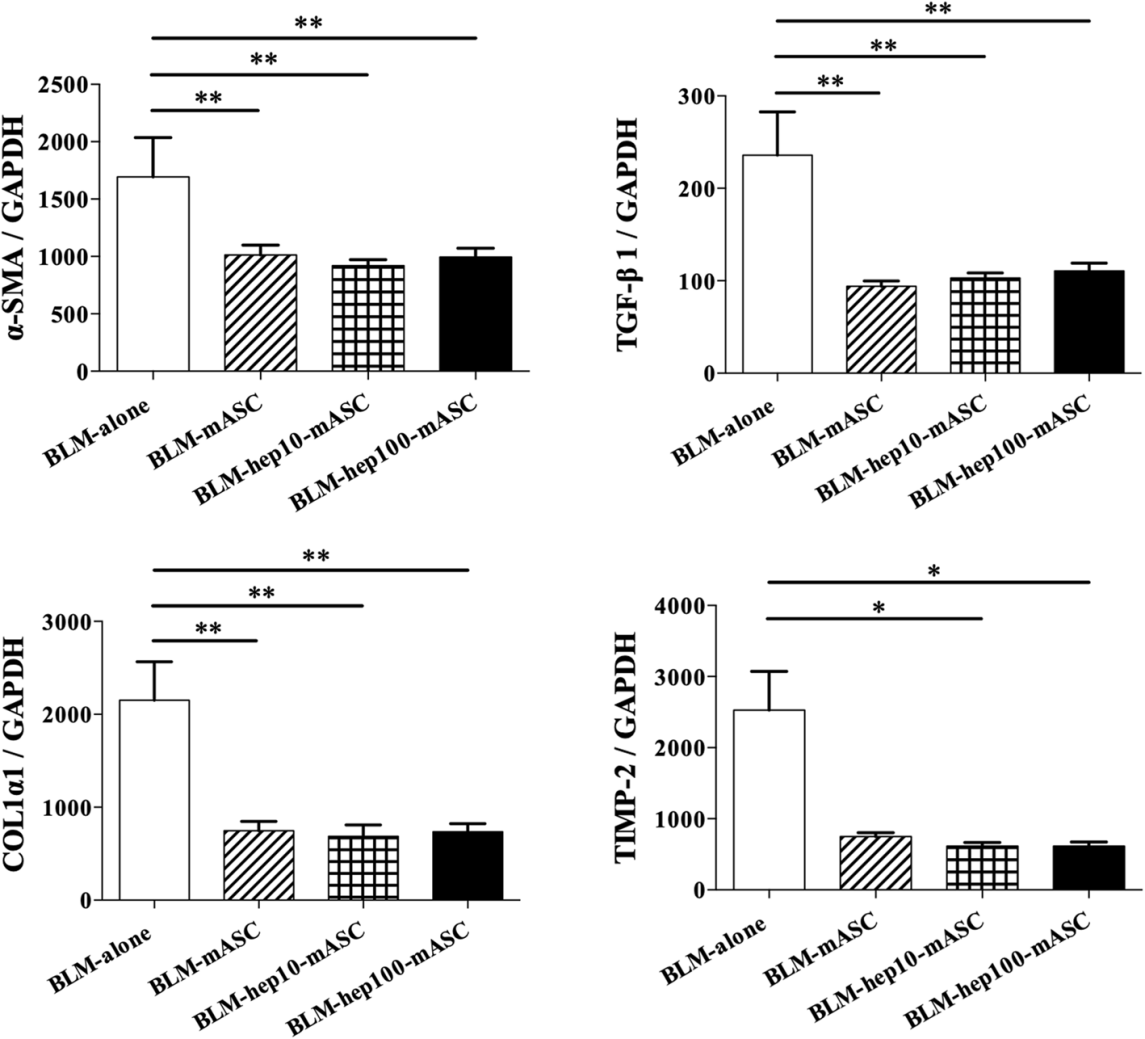
Genes related to fibrosis in skin were regulated by administering hep-mASCs. The skin mRNA expression levels of α-SMA, TGF-β1, and COL1α1 were significantly downregulated in the BLM-mASC group, the BLM-hep10-mASC group, and the BLM-hep100-mASC group when compared to those in the BLM-only group. The skin mRNA expression levels of TIMP-2 were significantly downregulated in the BLM-hep10-mASC and BLM-hep100-mASC groups when compared to those in the BLM-only group, but were not downregulated in the BLM-mASC group. *n* = 7 in each group. Data are shown as mean ± SEM. *P < 0.05; **P < 0.01 vs. BLM-alone group

To evaluate the anti-fibrotic effects of hep-mASCs, skin mRNA expression was analysed by qRT-PCR. The skin mRNA expression levels of α-SMA, TGF-β1, and COL1α1 were significantly downregulated in the BLM-mASC group, the BLM-hep10-mASC group, and the BLM-hep100-mASC group when compared to those in the BLM-only group. The skin mRNA expression levels of TIMP-2 were significantly downregulated in the BLM-hep10-mASC and BLM-hep100-mASC groups when compared to those in the BLM-only group, but were not downregulated in the BLM-mASC group (Fig. 6). There was no significant difference in the skin mRNA expression levels of GREM-1 between the groups (Additional file 2).

### Protein levels of Smad2/3 and phospho-Smad 2/3 in the skin of BLM-SSc mice were regulated by administering LMWH-activated mASCs

**Fig. 7:**
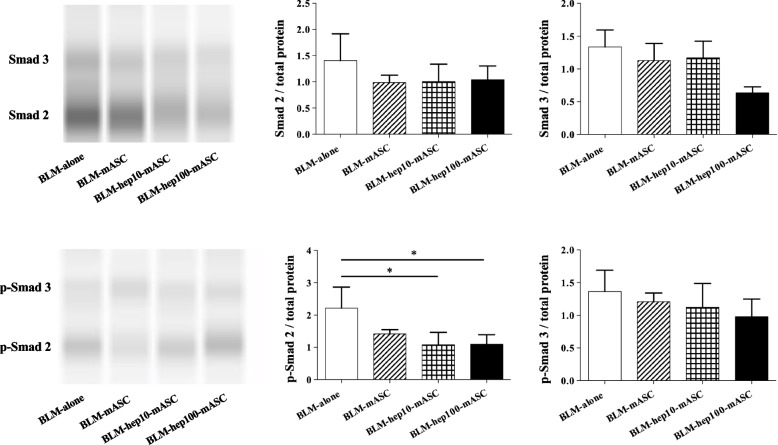
The protein levels of Smad 2/3 and p-Smad 2/3 in skin tissue. The protein levels of Smad 2/3 and p-Smad 2/3 in skin tissue were measured with western blotting. Compared to the BLM-only group, p-Smad 2 was significantly reduced in the BLM-hep10-mASC and BLM-hep100-mASC groups. *n* = 5 in each group. Data are shown as mean ± SEM. *P < 0.05 vs. BLM-alone group

To evaluate TGF-β1 activity in skin tissue, the protein levels of Smad 2/3 and phospho-Smad (p-Smad) 2/3 were measured using western blotting. Compared to the BLM-only group, p-Smad 2 was significantly reduced in the BLM-hep10-mASC and BLM-hep100-mASC groups, but was not reduced in the BLM-mASC group. In the BLM-mASC, BLM-hep10-mASC, and BLM-hep100-mASC groups, both Smad 2/3 and p-Smad 3 tended to be reduced in comparison with those in the BLM-alone group (Fig. 7).

## Discussion

Recently, Okamura et al. reported that skin fibrosis was suppressed after intravenous administration of mASCs to BLM-SSc models and sclerodermatous chronic graft-versus-host disease models [34]. A comparison of the therapeutic effects of systemic administration of human BM-MSCs and human ASCs in a mouse model, in which fibrosis was induced in the skin by hypochlorous acid, was also reported [35]. The ASCs significantly suppressed dermal thickening and downregulated the expression of mRNAs for inflammatory cytokines and factors involved in tissue remodelling (in the skin tissue) when compared with BM-MSCs. Regarding clinical application, Scuderi et al. administered autologous ASCs subcutaneously in either the face or the diseased parts of the limb in 6 patients with SSc and showed that the treatment reduced the thickness of the skin without causing any localised complications [36]. However, to the best of our knowledge, there are no reports on the systemic administration of ASCs to patients with SSc.

It is desired to improve the migration ability to the lesion sites, anti-inflammation effect, and anti-fibrosis effect of the administered ASCs. Systemic administration of ASCs is desirable to obtain their full effect against SSc. To obtain a sufficient therapeutic effect, it is desirable to administer a large number of ASCs. However, pulmonary embolism caused by the intravenous administration of ASCs has been reported [37], which hinders the development of this treatment. The ASCs from patients with SSc have reduced proliferative, metabolic, and chemotactic activities when compared with those from healthy individuals [38]. Furthermore, patients with SSc may not have enough fat due to nutritional disorders caused by gastrointestinal dysfunction [39]. If cell functions of ASCs could be enhanced, it would be possible to reduce the number of ASCs used for treatment and overcome the decrease in the number of ASCs due to underlying diseases. In addition, when the same number of cells is administered, ASCs with enhanced functions are considered to have a higher therapeutic effect than normal ASCs.

In the present study, LMWH enhanced HGF production from mASCs and the migration ability of mASCs and also promoted the mRNA expression of cell migration factors, such as SDF-1, CXCR4, and CXCR7. Because the migration ability of mASCs was significantly enhanced with 10 and 100 μg/mL of LMWH in vitro, the migration assay of mASCs in vivo was examined at 10 and 100 μg/mL of LMWH. The administered mASCs have the ability to migrate to the inflammatory site [40]. In the present study, mASCs accumulated in the skin lesions of BLM-SSc mice at a 10 μg/mL of LMWH. The higher accumulation of mASCs at the lesion site, which was enhanced by LMWH, was considered to be one of the reasons for the BLM-hep-mASC group to be more effective than the BLM-mASC group. The effect of hep-mASCs on BLM-SSc mice is considered to be the direct mechanism by which hep-mASCs accumulate in the skin. Furthermore, the effect of HGF released from hep-mASCs accumulated in the skin can be expected.

In this study, there was a discrepancy between the degree of accumulation of mASCs in skin lesions and the therapeutic effect. Although the reason is unknown, it is considered that hep100-mASCs are superior to hep10-mASCs in quality and quantity of cell functions, excluding migration ability and secretion of humoral factors for anti-inflammatory and anti-fibrotic effects. In addition, gene expression of the migration factors of mASCs was maximised in the hep100-mASC group, but the accumulation in skin lesions was maximised in the hep10-mASCs group. However, the gene expression and function of mASCs do not necessarily correlate linearly with cell migration ability; there are many other factors involved such as other chemokines and growth factors. It is possible that any of these may affect the enhancement of the migratory function of mASCs by LMWH. To investigate this further, it will be necessary to examine the effect of LMWH on mASCs in more detail in the future.

HGF activates the c-Met receptor, thereby activating the downstream PI3K/Akt and ERK1/2 pathways, and exerts anti-inflammatory effects by downregulating NF-κB signalling, decreasing IL-6 production, increasing IL-10 production, and promoting Treg differentiation [41, 42]. It also exerts anti-fibrotic effects by downregulating TGF-β signalling, suppressing fibroblasts, increasing matrix metalloproteinase 9, and decreasing tissue inhibitor of metalloproteinases 1 and 2 [43–45]. The efficacy of HGF on BLM-SSc mice and the enhancing anti-inflammatory and anti-fibrotic effects of BM-MSCs transfected with the *HGF* gene have been reported [45, 46].

TGF-β1 is involved in fibrosis through the Smad family molecules [47]. In this study, the protein levels of Smad 2/3 and p-Smad 2/3 in skin tissue were measured to evaluate TGF-β1 activity. The results showed that hep-mASCs significantly reduce p-Smad2 in the skin of BLM-SSc mice. This result suggests that HGF from mASCs, increased by LMWH, affects the inhibition of fibrosis by decreasing TGF-β1 activity and Smad signalling.

In the BLM-mASC group of this study, mASCs alone had no effect on skin fibrosis in BLM-SSc mice, whereas mRNA expression of fibrotic synthesis factors was suppressed in skin tissue. It is suspected that the necessary amount of mASCs did not accumulate in the skin. In preliminary experiments, it was confirmed that the therapeutic effects were obtained with 1.0 × 10^5^ mASCs, which is four times larger than the number of cells used in the present study (Additional file 3).

GREM-1 is a known pro-fibrotic molecule in SSc. It has been reported that IL-6 signalling increases GREM-1 expression, leading to fibrosis [48, 49]. IL-13 has been reported to be involved in fibrosis without GREM-1 [49]. Skin fibrosis is also considered to be mediated by Th2/Th17 immune polarity [50]. In this study, skin mRNA expression of IL-13 and IL-17 in the BLM-hep100-mASC group was significantly downregulated compared to the BLM-alone group, while GREM-1 and IL-6 were unchanged. These results suggest that one of the mechanisms by which hep-mASCs inhibit fibrosis is the correction of Th2/Th17 immune polarity, which is not mediated by IL-6 or GREM-1.

One limitation of this study was that we validated it in only one model using one strain of SSc. In the future, the study should be extended to include other strains as well, such as tight-skin mice.

## Conclusions

Based on the results of this study, hep-mASCs appear to have higher anti-inflammatory and anti-fibrotic effects than mASCs alone and may be a promising candidate for the treatment of SSc in the future. The results of this study showed a possible method to reduce both the number of ASCs administered and the risk of pulmonary embolism, as well as to enhance therapeutic effects. Development of methods to further enhance the cellular functions of ASCs is required.

## Supplementary Information

**Additional file 1.** Experimental protocol. For induction of skin fibrosis, 8-week-old female Balb/c mice were subcutaneously injected with 100 μg/100 μL of bleomycin daily for 3 weeks. mASCs were administered 1 week after the start of bleomycin administration and evaluated at 3 weeks. mASCs: mouse adipose-derived mesenchymal stem cells.

**Additional file 2.** Experimental data. Genes of GREM-1 and IL-6 were not affected by hep-ASCs. For the skin mRNA expression levels of gremlin-1 (GREM-1), and interleukin (IL)-6 which may induce GREM-1, there was no significant difference between the groups. n = 7 in each group. Data are shown as mean ± SEM.

**Additional file 3.** Experimental data. Skin fibrosis reduced after administering mASCs. 1×10^5^ mASCs administration significantly reduced dermal thickness (distance between epidermal–dermal junction and dermal–fat junction) and hydroxyproline content. n = 6 in each group. Data are presented as mean ± SEM. **P < 0.01. ***P < 0.005 vs. BLM-alone group.

## Data Availability

The datasets used and/or analysed during the current study are available from the corresponding author on reasonable request.

## Abbreviations

SSc: Systemic sclerosis
MSCs: Mesenchymal stem cells
ASCs: Adipose-derived mesenchymal stem cells
mASCs: Mouse ASCs
LMWH: Low-molecular-weight heparin
hep-mASCs: mASCs activated with LMWH
HGF: Hepatocyte growth factor
BLM: Bleomycin
PBS: Phosphate-buffered saline
DAPI: 4’,6-Diamidino-2-phenylindole
SDF-1: Stromal derived factor-1
CXCR-4: C-X-C chemokine receptor (CXCR) type 4
CXCR-7: CXCR type 7
IL-2: Interleukin-2
IL-4: Interleukin-4
IL-6: Interleukin-6
IL-10: Interleukin-10
IL-13: Interleukin-13
IL-17: Interleukin-17
INF-γ: Interferon-gamma
α-SMA: α-smooth muscle actin
TGF-β1: Transforming growth factor β1
COL1α1: Collagen type 1 alpha 1
TIMP-2: Tissue inhibitor of metalloproteinase 2
GREM-1: Gremlin-1
GAPDH: Glyceraldehyde 6-phosphate dehydrogenase
p-Smad: Phospho-Smad
