# Supplementary material for: Therapeutic effects of adipose-derived mesenchymal stem/stromal cells with enhanced migration ability and hepatocyte growth factor secretion by low-molecular-weight heparin treatment in bleomycin-induced mouse models of systemic sclerosis
Source: Arthritis Res Ther. 2022 Oct 7;24:228. doi: 10.1186/s13075-022-02915-6 (PMC9540693; doi:10.1186/s13075-022-02915-6)
Supplement: Supplementary file 1 — Additional file 1. Experimental protocol. For induction of skin fibrosis, 8-week-old female Balb/c mice were subcutaneously injected with 100 μg/100 μL of bleomycin daily for 3 weeks. mASCs were administered 1 week after the start of bleomycin administration and evaluated at 3 weeks. mASCs: mouse adipose-derived mesenchymal stem cells. [file 13075_2022_2915_MOESM1_ESM.docx]

Additional file 1

**SUPPLEMENTRY METHOD**

Additional file 1


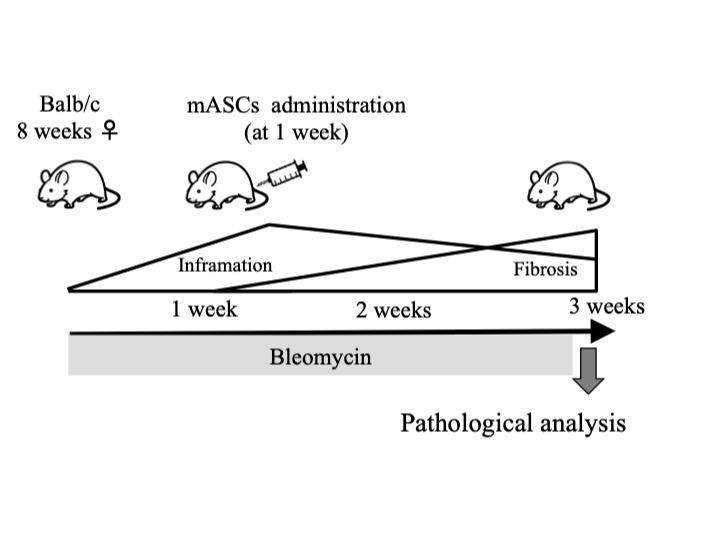
Experimental protocol. For induction of skin fibrosis, 8-week-old female Balb/c mice were subcutaneously injected with 100 μg/100 μL of bleomycin daily for 3 weeks. mASCs were administered 1 week after the start of bleomycin administration and evaluated at 3 weeks. mASCs: mouse adipose-derived mesenchymal stem cells
