# Supplementary material for: Therapeutic effects of adipose-derived mesenchymal stem/stromal cells with enhanced migration ability and hepatocyte growth factor secretion by low-molecular-weight heparin treatment in bleomycin-induced mouse models of systemic sclerosis
Source: Arthritis Res Ther. 2022 Oct 7;24:228. doi: 10.1186/s13075-022-02915-6 (PMC9540693; doi:10.1186/s13075-022-02915-6)
Supplement: Supplementary file 2 — Additional file 2. Experimental data. Genes of GREM-1 and IL-6 were not affected by hep-ASCs. For the skin mRNA expression levels of gremlin-1 (GREM-1), and interleukin (IL)-6 which may induce GREM-1, there was no significant difference between the groups. n = 7 in each group. Data are shown as mean ± SEM. [file 13075_2022_2915_MOESM2_ESM.docx]

Additional file 2

**SUPPLEMENTRY DATA**

Additional file 2


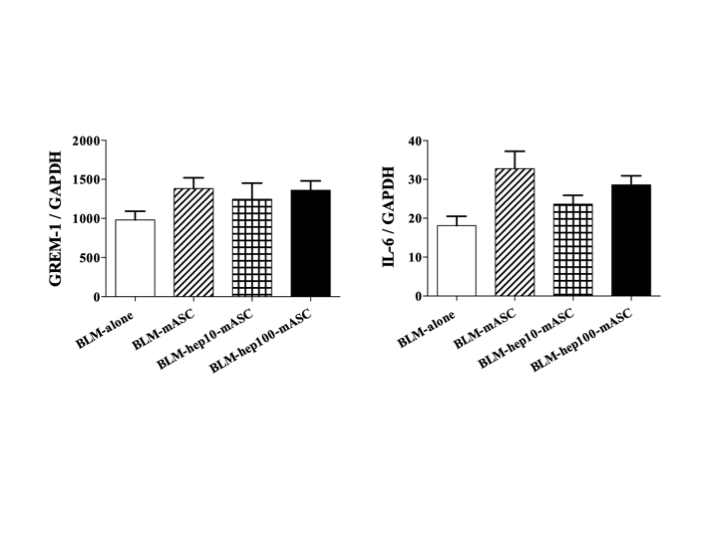


Genes of GREM-1 and IL-6 were not affected by hep-ASCs. For the skin mRNA expression levels of gremlin-1 (GREM-1), and interleukin (IL)-6 which may induce GREM-1, there was no significant difference between the groups. n = 7 in each group. Data are shown as mean ± SEM.
