# Supplementary material for: Therapeutic effects of adipose-derived mesenchymal stem/stromal cells with enhanced migration ability and hepatocyte growth factor secretion by low-molecular-weight heparin treatment in bleomycin-induced mouse models of systemic sclerosis
Source: Arthritis Res Ther. 2022 Oct 7;24:228. doi: 10.1186/s13075-022-02915-6 (PMC9540693; doi:10.1186/s13075-022-02915-6)
Supplement: Supplementary file 3 — Additional file 3. Experimental data. Skin fibrosis reduced after administering mASCs. 1×10^5^ mASCs administration significantly reduced dermal thickness (distance between epidermal–dermal junction and dermal–fat junction) and hydroxyproline content. n = 6 in each group. Data are presented as mean ± SEM. **P < 0.01. ***P < 0.005 vs. BLM-alone group. [file 13075_2022_2915_MOESM3_ESM.docx]

Additional file 3

**SUPPLEMENTRY DATA**

Additional file 3


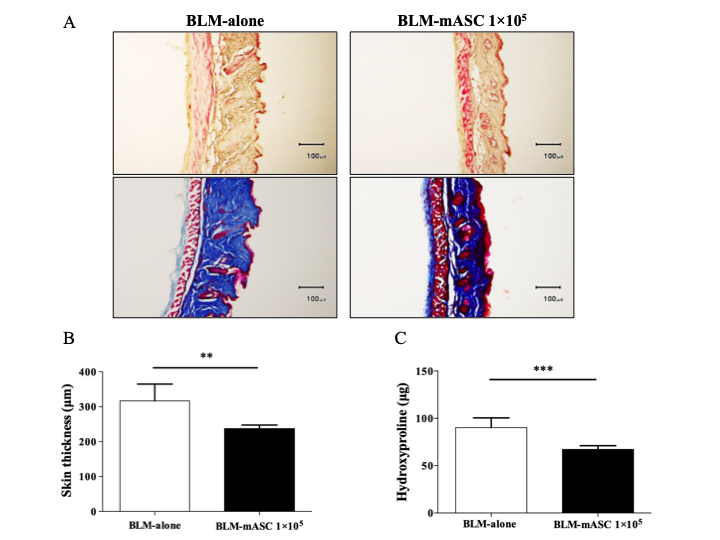


Skin fibrosis reduced after administering mASCs. 1×105 mASCs administration significantly reduced dermal thickness (distance between epidermal-dermal junction and dermal-fat junction) and hydroxyproline content. n = 6 in each group. Data are presented as mean ± SEM. **P < 0.01. ***P < 0.005 vs. BLM-alone group.
